# The Direction of Stretch-Induced Cell and Stress Fiber Orientation Depends on Collagen Matrix Stress

**DOI:** 10.1371/journal.pone.0089592

**Published:** 2014-02-24

**Authors:** Abhishek Tondon, Roland Kaunas

**Affiliations:** Department of Biomedical Engineering, Texas A&M University, College Station, Texas, United States of America; University of California, Berkeley, United States of America

## Abstract

Cell structure depends on both matrix strain and stiffness, but their interactive effects are poorly understood. We investigated the interactive roles of matrix properties and stretching patterns on cell structure by uniaxially stretching U2OS cells expressing GFP-actin on silicone rubber sheets supporting either a surface-adsorbed coating or thick hydrogel of type-I collagen. Cells and their actin stress fibers oriented perpendicular to the direction of cyclic stretch on collagen-coated sheets, but oriented parallel to the stretch direction on collagen gels. There was significant alignment parallel to the direction of a steady increase in stretch for cells on collagen gels, while cells on collagen-coated sheets did not align in any direction. The extent of alignment was dependent on both strain rate and duration. Stretch-induced alignment on collagen gels was blocked by the myosin light-chain kinase inhibitor ML7, but not by the Rho-kinase inhibitor Y27632. We propose that active orientation of the actin cytoskeleton perpendicular and parallel to direction of stretch on stiff and soft substrates, respectively, are responses that tend to maintain intracellular tension at an optimal level. Further, our results indicate that cells can align along directions of matrix stress without collagen fibril alignment, indicating that matrix stress can directly regulate cell morphology.

## Introduction

Cyclic stretching causes the alignment of several cell types perpendicular to the direction of stretch [1]–[3] with the extent of alignment dependent on stretch amplitude, frequency and spatial pattern [4]–[6]. These experiments are generally performed with cells cultured on silicone rubber sheets coated with matrix proteins (typically collagen type-I or fibronectin). On these substrates, cells contain actin stress fibers (SFs) that generate isometric tension balanced by forces in the substrate [7]. Experiments supported by theoretical models indicate that disruption of this mechanical equilibrium by cyclic stretch causes cells and their SFs to align perpendicular to the direction of strain in effort to reestablish tensional homeostasis [8], [9]. Inhibition of actomyosin contractility using inhibitors of the Rho GTPase and myosin light-chain kinase pathways suppress SF formation in the central and peripheral regions, respectively, with any remaining SFs orienting parallel to the stretch direction [5].

Experiments involving cells cultured on soft hydrogels have demonstrated that substrate stiffness strongly regulates many cell processes, including cell–cell adhesion [10], [11], cell–substrate adhesion [12], and cell differentiation [13]. The extents of cell spreading and SFs formation in endothelial cells and fibroblasts increase with increasing hydrogel stiffness, showing a sharp transition at a stiffness of ∼3 kPA [14]. The extent of spreading of mesenchymal stem cells measured on very soft hydrogels (∼1 kPa) shows that cells spread little on thick gels, but below a threshold thickness of ∼20 µm the cells spread increasingly more as the gel thickness decreases [15]. Finite element modeling of gel deformation by contractile cells predicts that matrix strain rapidly decays with distance from the cell edge, with a characteristic distance of 10 µm [16]. These studies indicate that cells perceive very thin gels as having a stiffness approaching that of the material supporting the gel since the supporting material constrains cell-induced matrix deformation.

Since the stiffness of silicone rubber (on the order of MPa [6]) is well above the range that cells can deform via contractile forces, we investigated how cells respond to stretching on soft hydrogels (on the order of tens of Pa [17] ). Quinlan et al. [18] recently reported that stretch-induced alignment is attenuated in cells seeded on soft polyacrylamide, though they did not suggest a mechanism. Given that the direction cells align when stretched on silicone rubber depends on actomyosin contractile activity and contractile activity is low in cells on soft hydrogels, we postulated that stretching cells on a soft substrate would induce cell and SF alignment parallel to the direction of stretch in a manner dependent on substrate stiffness and actomyosin contractile activity.

## Materials and Methods

### Cell Culture

U2OS osteosarcoma cells stably expressing GFP-actin (MarinPharm GmbH, Germany) were cultured in DMEM (Gibco) supplemented with 10% fetal bovine serum (Gibco), 2 mM L-glutamine (HyClone), 1 mM sodium pyruvate (HyClone) and 1 mM (HyClone) penicillin/streptomycin in a humidified 5%CO_2_/95% air incubator as described previously [1].

### Collagen Hydrogel Preparation and Stretching Experiment

Silicone rubber stretch chambers (Strex, Japan) were modified to form a circular well (15 mm diameter) by adhering a silicone rubber sheet onto the chambers (Fig. S1). The chambers were initially coated with collagen (4 µg/cm^2^) by incubating 100 µl of 0.3 mg/ml rat tail collagen type-I (BD Biosciences) in the well and allowing the solution to evaporate. The collagen solution (3 mg/ml) was then added to form a gel within the collagen-coated well as described previously [19].

Cells were cultured on the top surface of the collagen gels and subjected to cyclic stretch by stretching the chambers with two linear motors (Zaber, Canada) as described previously [4]. The entire stretch apparatus was mounted on the stainless steel stage (Gibraltar) of a Nikon FN1 upright microscope housed in a custom-made acrylic enclosure maintained at 37°C using a heat gun (Omega) regulated by a temperature controller (Omega).

### Three-point Finite Strain Analysis

The strain fields produced by the device were determined by tracking the displacement of markers on the collagen gel and silicone rubber surfaces. Strains on the bottom surface of the silicone rubber sheeting were measured by marking membranes at several points with a permanent marker and imaging before and after stretch using nominal stretch values ranging from 2.5 to 12.5%. To quantify the strains on the collagen gel surface, red fluorescent beads (0.2 µm Fluospheres, Molecular Probes) were mixed into the gel prior to polymerization to serve as fiducial markers. Triads of markers in a focus plane in various locations on the surface were selected to compute the symmetric Lagrangian strain tensor at each location. The finite strains in the longitudinal (*E*_1_) and lateral directions (*E*_2_) were computed from the *E*_11_ and *E*_22_ components of the Lagrangian strain tensor [20] using Eqns. 1A and 1B.
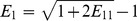
(1A)




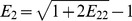
(1B)


### Quantification of SF and Collagen Fibril Organization

After stretching, cells were rinsed with PBS, fixed with 4% paraformaldehyde and stained with Alexa 488-phalloidin (Molecular Probes) as described previously [21]. Although the cells expressed GFP-actin, Alexa 488-phalloidin staining provides a stronger signal for SF visualization that resists photobleaching. Images were captured using a Nikon C1 laser scanning confocal head with a 60X water-dipping objective illuminated with a 40-mW Argon ion laser and green Helium Neon laser (Melles Griot). Collagen fibrils were imaged by confocal reflectance. The images were analyzed using a custom algorithm in MATLAB (the MathWorks, Natick, MA) to quantify the density distribution 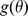 of SFs within each cell as previously described [1] or for the entire field of view for collagen fibrils. The density distributions from multiple cells were summarized with angular histograms. An order parameter
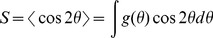
(2)was calculated for each cell to quantify the extent of alignment. Values of 1 or −1 indicate alignment parallel or perpendicular to the stretch direction, respectively, while a value of 0 indicates no alignment.

### Quantification of Cell Alignment

The shape of each cell was determined from the background signal of the Alexa 488-phalloidin stained cells. Each cell was fit to an equivalent ellipse using NIH ImageJ software [22] to quantify cell orientation 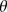 and order parameter 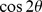.

### Live Microscopy

One hour before starting the experiment, the media in stretch chamber was changed to Hyclone L-15 CO_2_-independent media (Hyclone). The stretch device was mounted under the objective of the confocal microscope and subjected to a 10% step change in stretch with images of GFP-actin captured at 10 min intervals.

### Statistical Analysis

Significant differences in order parameters between groups were identified using ANOVA followed by Student-Newman–Keuls posthoc multiple comparisons testing.

## Results

### Strain Measurements

**Figure 1:**
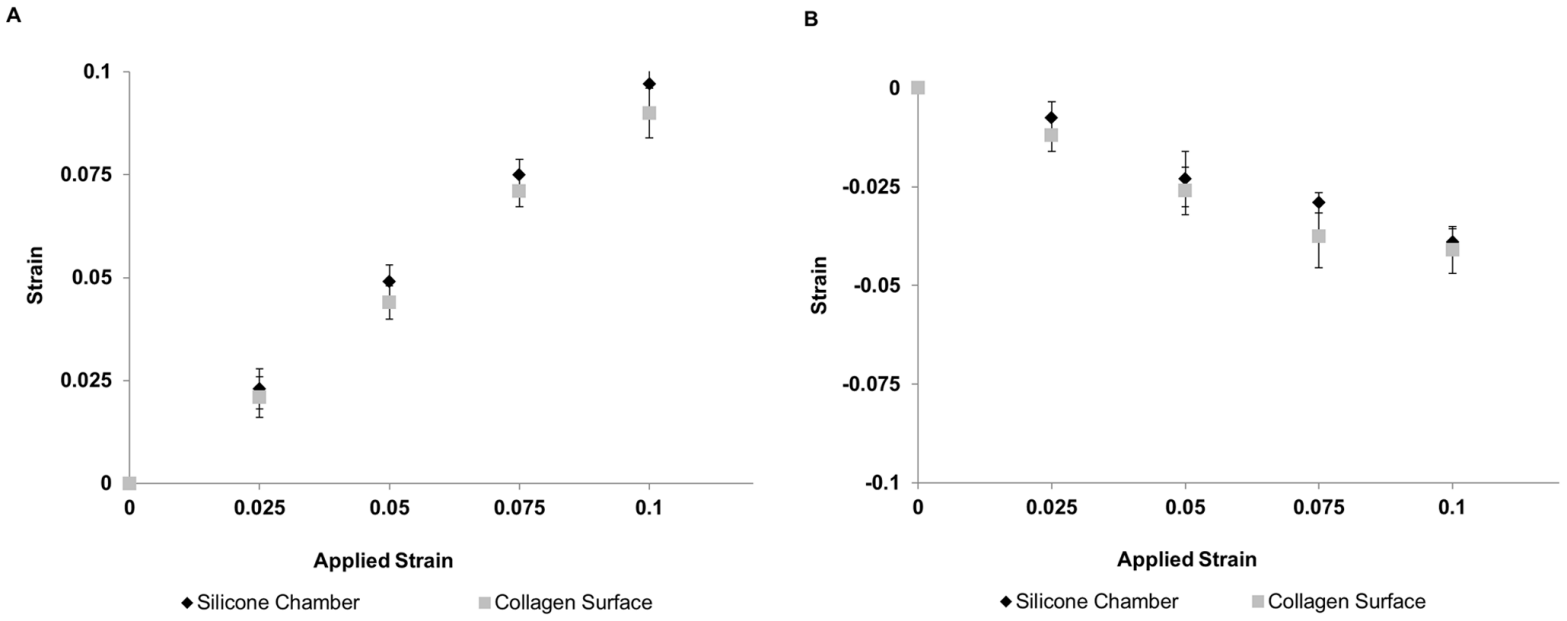
Strain is efficiently transmitted to the collagen gel surface. The longitudinal (A) and lateral (B) strains (mean ± SEM; n = 6) within the region of the silicone rubber chamber used to support the collagen gels were quantified at the surfaces of the silicone rubber sheet (black) and the collagen gel (grey).

Two-dimensional strains measured on the surfaces of 500 µm-thick collagen gels and the supporting silicone rubber membranes were very similar (Fig. 1). Strains parallel and perpendicular to the principal stretch direction on surface of collagen for a nominal stretch of 10% were 0.093±0.009 and −0.040±0.01 (Fig. 1A and 1B), respectively, resulting in a Poisson’s ratio of 0.43±0.01 (mean±SD). Strains observed on the surface of silicone rubber membranes were 0.097±0.01 and −0.046±0.005 parallel and perpendicular to that of principal stretch direction, respectively, resulting in a Poisson’s ratio of 0.47±0.05 (mean±SD).

### Cyclic Stretch-induced SF Alignment Depend on the Thickness of the Collagen Gel

**Figure 2:**
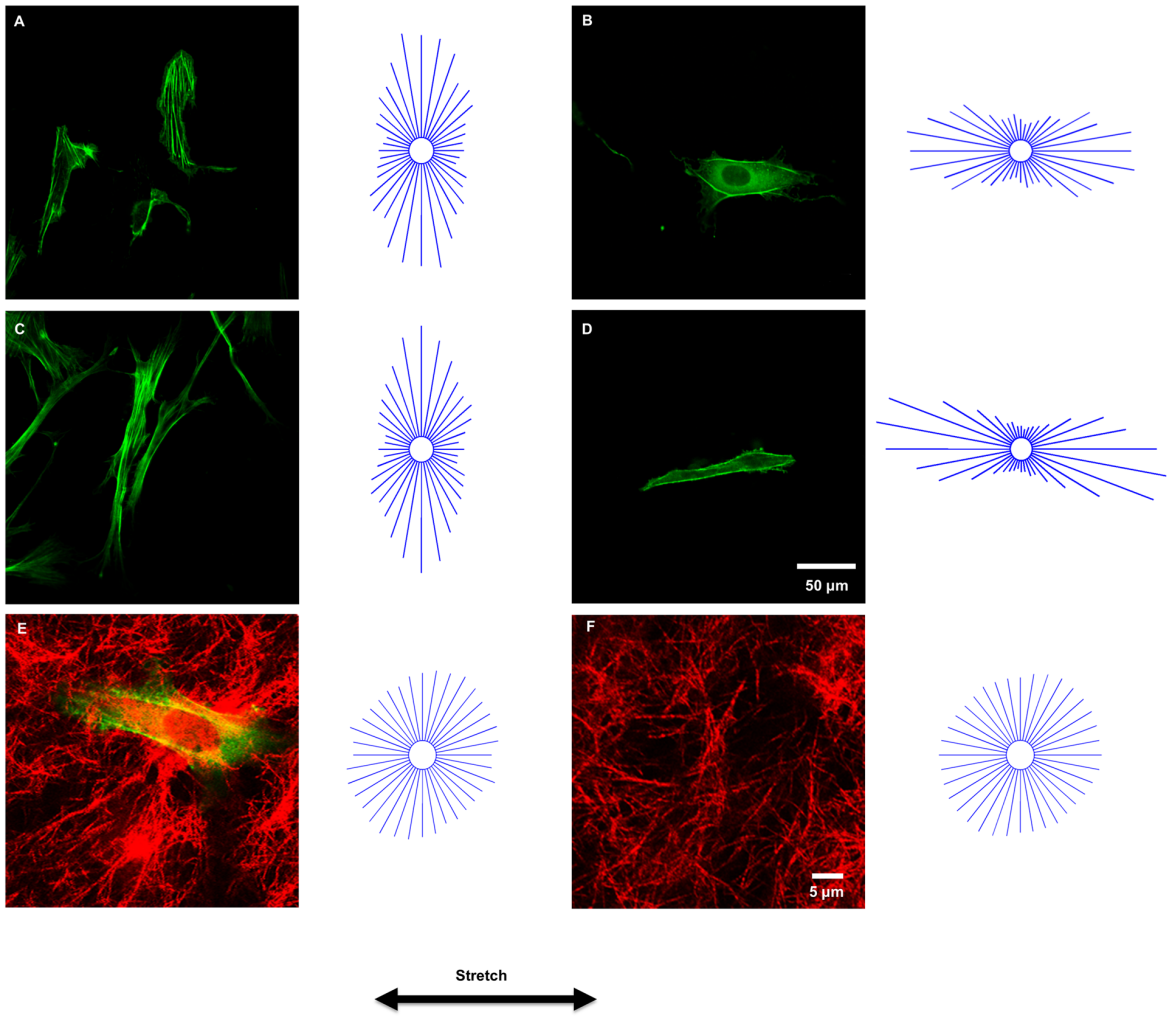
Cyclic stretch-induced SF alignment on soft collagen gels and stiff silicone rubber sheets. A–D: Representative images and circular histograms depicting SF angular distributions of non-confluent U2OS cells (A, B) (n = 90 for each condition) and hMSCs (C, D) (n = 60 for each condition) subjected to 3 h of 10% cyclic uniaxial stretch at frequency of 1 Hz on collagen-coated rubber sheets (A, C) and collagen gels (B, D). Scale bar, 50 µm. E, F: Representative confocal reflectance images of collagen fibrils (red) in regions containing a U2OS cell (E) and devoid of cells (F) and circular histograms depicting collagen fibril alignment after 3 h of 10% cyclic stretching at 1 Hz.; Scale bar, 5 µm.

We evaluated the effects of stretch on SF organization in non-confluent U2OS cells adhered onto the top of collagen gels. For comparison, experiments were also performed where the cells were adhered on the supporting silicone rubber membranes, but coated with a low concentration of collagen (4 µg/cm^2^) rather than the thick gel. In each case, the cells were subjected to 3 h of 10% cyclic uniaxial stretch at 1 Hz. Consistent with our previous findings using non-confluent and confluent U2OS and bovine aortic endothelial cells on fibronectin-coated silicone rubber [4], [5], [9], the SFs in cells on collagen-coated silicone rubber oriented perpendicular to the direction of stretch (Fig. 2A). In contrast, the cells and their SFs reoriented parallel to the direction of stretch on thick collagen gels (Fig. 2B). To determine if the result was cell type –specific, the experiments were repeated using human mesenchymal stem cells (Figs. 2C and D). Confocal reflectance images of collagen fibers in regions containing a cell (Fig. 2E) and regions devoid of cells (Fig. 2F) indicated that collagen fibrils did not co-align with the cells in response to cyclic stretching.

### Cells as well as Their SFs Reorient in a Cyclic Stretch Frequency-dependent Manner

**Figure 3:**
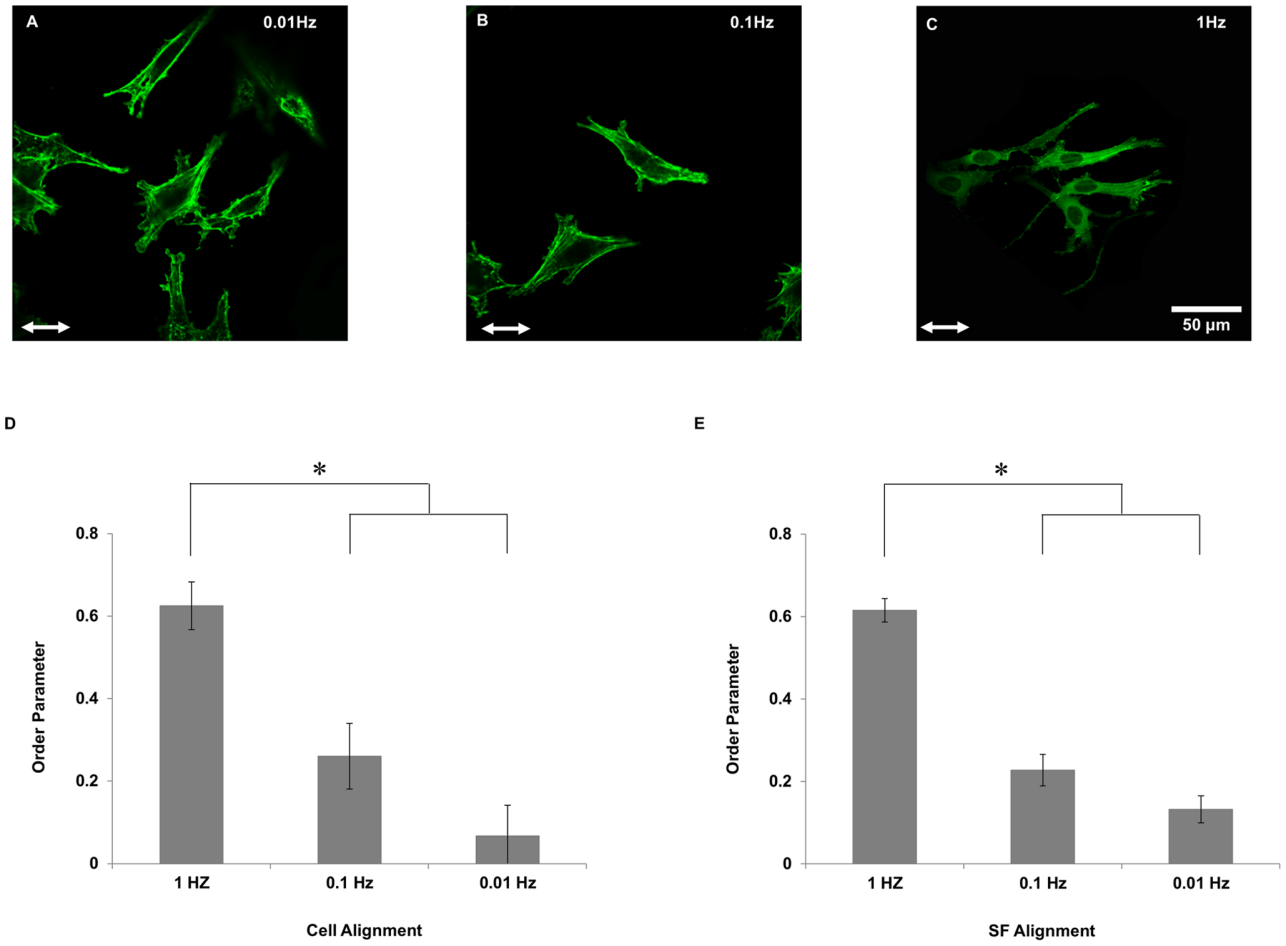
Cyclic stretch-induced cell and SF alignment on soft collagen gels depends on stretch frequency. Representative images of non-confluent U2OS cells adhered on a soft collagen matrix subjected to 3 h of 10% cyclic stretch at frequencies of 0.01 (A), 0.1 (B) and 1 Hz (C). Order parameters for cells (D) and SFs (E) were computed for each cell to quantify the extents of alignment and the results were summarized (mean ± SEM; n = 90). * indicates significant differences between groups as determined by ANOVA followed by Student-Neuman–Keuls post-hoc multiple comparison testing (P<0.01). Scale bar, 50 µm.

To determine the dependence on stretch frequency on thick collagen gels, the extent of cell and SF alignment was quantified in U2OS cells subjected to 3 h of 10% cyclic uniaxial stretch at 0.01, 0.1 and 1 Hz on collagen gels. At 0.01 Hz, there was no cell or SFs alignment in any direction (Figs. 3A, D and E). Increasing the frequency of stretch to 1 Hz significantly increased alignment parallel to the stretch direction (Figs. 3C, D and E), while stretching at 0.1 Hz had an intermediate response (Figs. 3B, D and E). These results were consistent with the frequency-dependence we previously observed when stretching endothelial and U2OS cells on fibronectin-coated silicone rubber sheets [1], [5].

### Cells and Their SFs Align in Response to Step Stretch on Cells Adhered Onto Thick Collagen Gels, but not on Collagen-coated Silicone Rubber Sheets

**Figure 4:**
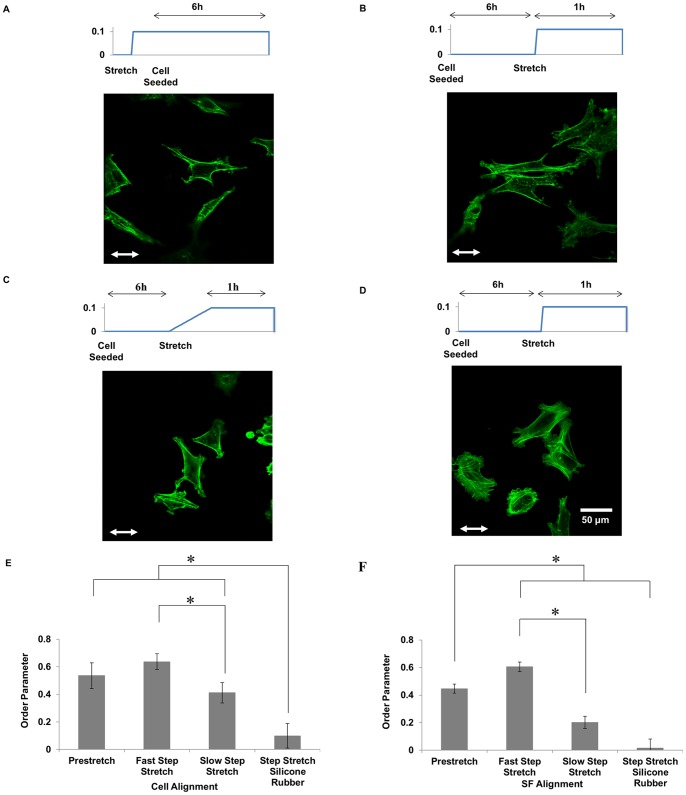
Effects of pre-stretch and strain rate on steady stretch-induced cell and SF alignment. Representative images of non-confluent U2OS cells seeded on 10% pre-stretched collagen gel (A) or adhered onto collagen gels (that were not pre-stretched) subjected to 10% stretch at ramp rates of 20%/s (B) and 0.2%/s (C). Representative image of U2OS cells adhered onto collagen-coated silicone rubber sheets subjected to 10% stretch at 20%/s (D). Cell (E) and SF (F) order parameters (n = 90) are summarized. * indicates significant differences between groups as determined by ANOVA followed by Student-Neuman–Keuls post-hoc multiple comparison testing (P<0.01). Scale bar, 50 µm.

Gavara et al. [17] reported that collagen type-I hydrogels (1.45 mg/ml) have a stiffness of 23 Pa that increased to 137 Pa in response to a step equibiaxial stretch of 11%. Previous studies indicate that uniaxial stretching a collagen gel causes anisotropic changes in gel stiffness, with the stiffness increasing in the direction of stretching [23], [24]. To determine if anisotropic changes in gel stiffness contribute to cell and SF alignment, U2OS cells were cultured on collagen gels that were subjected to 10% uniaxial pre-stretch prior to cell attachment (Fig. 4A). After 6 h, there was significant cell (Fig. 4E) and SF (Fig. 4F) alignment parallel to the direction of matrix stretching. In contrast, there was no alignment observed in cells cultured on pre-stretched collagen-coated silicone rubber (data not shown). Next, we quantified the effects of applying the stretch after the cells had spread. A rapid stretch of 20%/s (Fig. 4B) resulted in an apparent increase in cell alignment (Fig. 4E) and a significant increase in SF alignment (Fig. 4F) relative to that induced by seeding cells on a pre-stretched gel. A slow stretch at 0.2%/s (Fig. 4C) induced significantly less cell and SF alignment than both the pre-stretch and rapid stretch treatments (Figs. 4E–F). Interestingly, a rapid stretch applied to cells on collagen-coated silicone rubber (Fig. 4D) did not induce any alignment (Figs. 4E–F).

### Extent of Cell and SF Alignment Depends on the Duration of Transient Step Stretch

**Figure 5:**
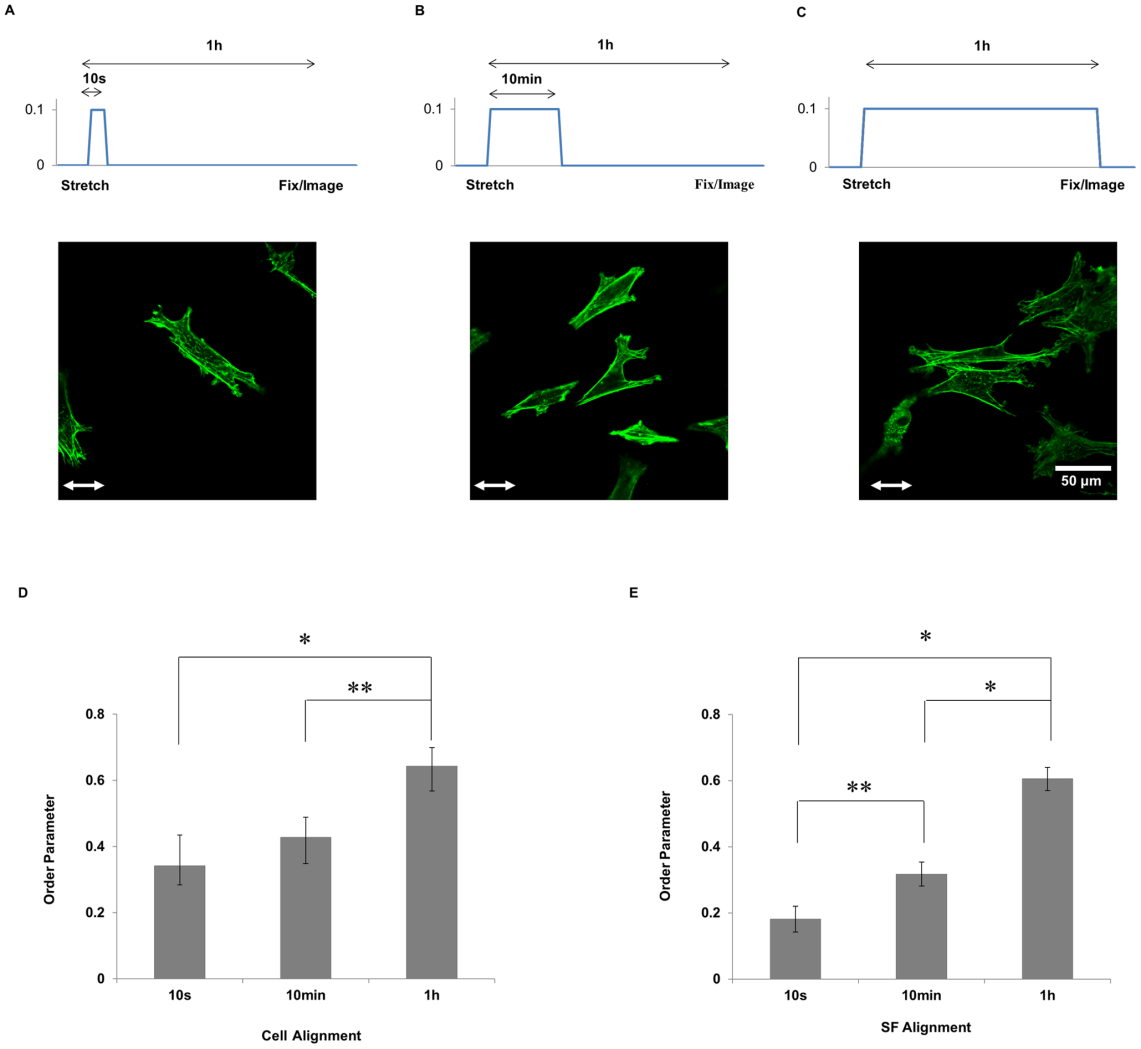
Extent of cell and SF alignment depends on the duration of transient step stretch. Representative images of non-confluent U2OS cells adhered onto collagen gels subjected to 10% transient step stretch, i.e. a regimen consisting of rapid ramp increase in stretch, a hold, and subsequent release of the stretch. The collagen gels were subjected to 10% stretch and held for 1 s (A), 10 min (B) and 1 h (C). Cell (E) and SF (F) order parameters (n = 90) are summarized. Significant differences between groups were determined by ANOVA followed by Student-Neuman–Keuls post-hoc multiple comparison testing (* = P<0.01, ** = P<0.05). Scale bar (A–C), 50 µm.

To assess the effects of the duration of stretching, we subjected the cells adhered on collagen gels to 10% transient step stretch, i.e. a regimen consisting of a rapid ramp increase in stretch (20%/s), a transient hold (10 s, 10 min or 1 h), and subsequent release of the stretch (Figs. 5A–C). In each case, the cells were fixed after a total elapsed time of 1 h. No alignment of cell or SFs occurred in response to 10 s of transient stretch (Figs. 5D and E). There was significantly more cell and SFs alignment in response to 1 h of transient stretch, while 10 min of transient stretch resulted in an intermediate response.

### Role of MLCK in SF Formation and Reorientation

**Figure 6:**
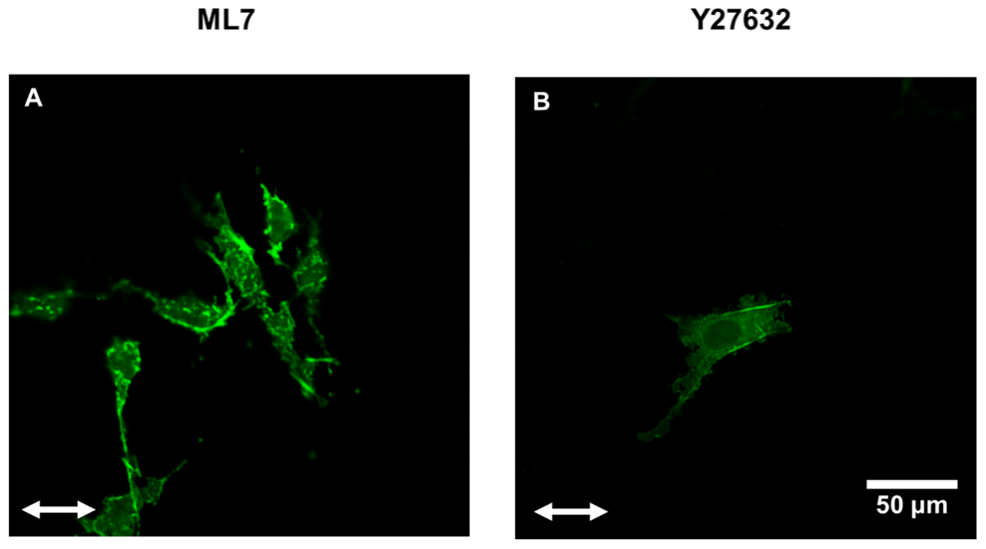
Roles of Rho-kinase and MLCK on cyclic stretch-induced SF alignment in cells on 3-D collagen gels. Representative images of non-confluent U2OS cells (n = 60) adhered on soft collagen gels subjected to 3 h of 10% cyclic uniaxial stretch at 1 Hz after treatment with 30 µM ML7 (A) or 10 µM Y27632 (B). Scale bar, 50 µm.

Consistent with previous findings with NIH 3T3 fibroblasts on soft polyacrylamide gels [14], we observed that SFs were less prevalent in cells on soft collagen hydrogels as compared to cells on stiff collagen-coated silicone rubber sheets (cf. Figs. 2B vs. 2A). Further, the few SFs observed in cells adhered onto soft collagen gels were primarily located in the cell periphery (cf. Figs. 2B and 2D). In contrast, both peripheral and central SFs were observed in cells on collagen-coated silicone rubber (cf. Figs. 2A and 2C). We have previously shown that Rho-kinase and myosin light-chain kinase (MLCK) regulate different populations of SFs: peripheral SFs are sensitive to MLCK inhibition, while central SFs are sensitive to Rho-kinase inhibition [5]. To assess the involvement of MLCK and Rho-kinase pathways in stretch induced SF alignment on cells adhered to collagen gels, we treated the U2OS cells with inhibitors of either MLCK (ML7) or Rho-kinase (Y27632) and subjected them to 10% cyclic stretch at 1 Hz for 3 h (Fig. 6). The cells were treated with either 10 µM Y27632 or 30 µM ML7 for 30 min prior to initiating stretch with the drug remaining in the culture media throughout the experiment. In cells treated with ML7, SFs were completely attenuated (Fig. 6A). In contrast, there was some reduction in the number of SFs in cells treated with Y27632, but these remaining fibers oriented roughly parallel to the direction of cyclic stretch (Fig. 6B).

### Dynamics of Stretch-induced SF Reorientation

**Figure 7:**
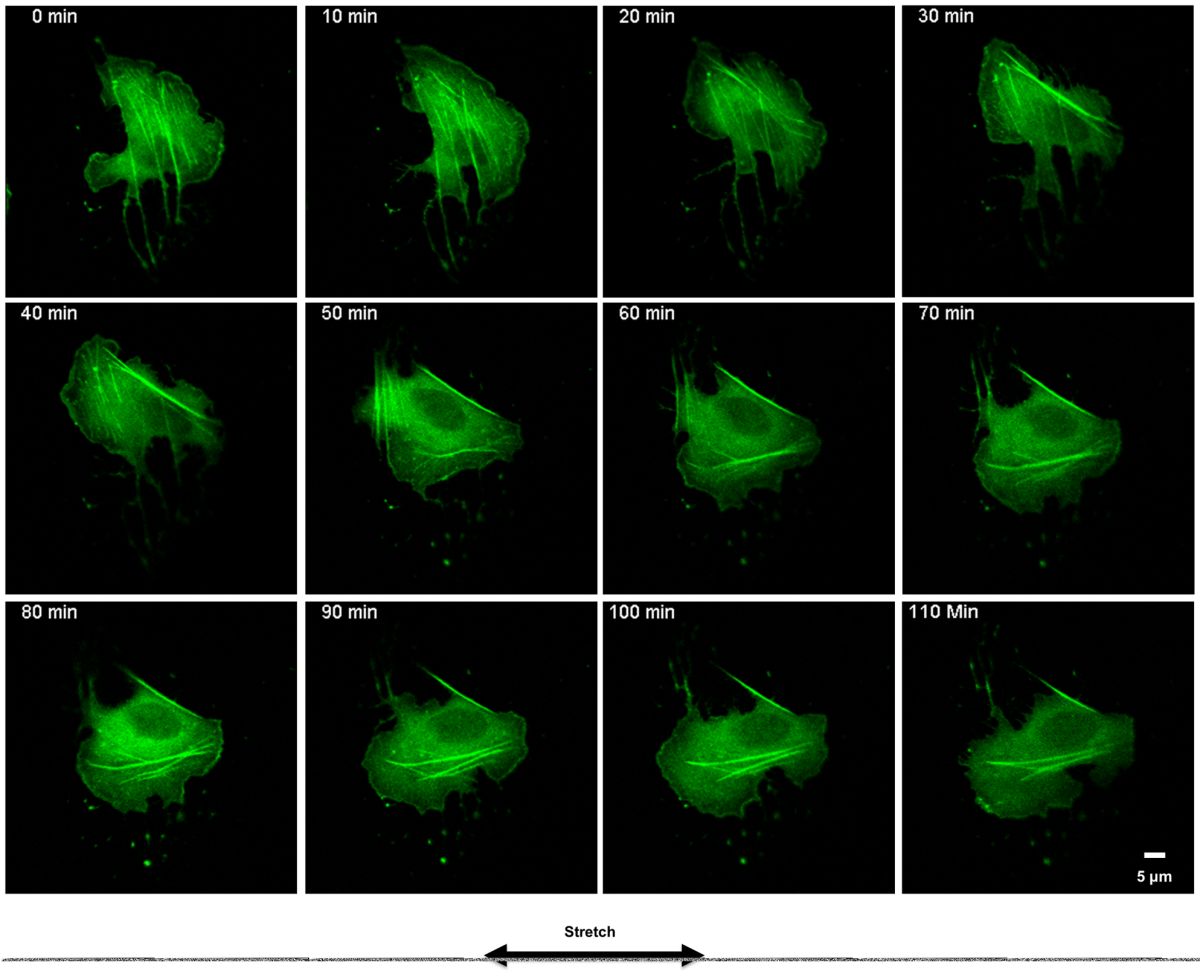
Live Cell Microscopy. Time-lapse images of a U2OS cell expressing GFP-actin subjected to subjected to 10% stretch at ramp rates of 20%/s. Imaging began immediately after the collagen hydrogel was stretched, with subsequent images captured at 10 min intervals for 2 h. Scale bar, 5 µm.

To observe the dynamic process of SF alignment in cells stretched on collagen gels, we collected time-lapse videos of GFP-labeled actin in U2OS cells. Figure 7 and Video S1 illustrate the evolution of SF reorganization in a representative cell subjected to 10% step uniaxial stretch. Initially, the cell contained SFs that were oriented roughly perpendicular to the axis of stretching. SFs oriented perpendicular to the direction of stretch began to disassemble after approximately 20 min, followed by the formation of new SFs primarily oriented in the direction of stretch. The experiment was repeated for multiple cells (data not shown) and similar responses were observed.

## Discussion

Our results demonstrate that stretch-induced cell and SF alignment are highly dependent on the mechanical properties of the collagen matrix upon which cells are cultured. Cyclic stretch promoted alignment parallel to the direction of stretch (cf. Figs. 2B and 2D) in cells with attenuated contractility caused by adhesion to a soft collagen gel, as judged by the relatively few SFs relative to that in the same cell type on collagen-coated silicone rubber. This is consistent with previous studies performed with cells on fibronectin-coated silicone rubber showing that stretching promotes SF alignment parallel to the direction of stretch when cell contractility is attenuated with small molecule inhibitors of Rho-kinase or MLCK [5], [21]. This is in stark contrast to the perpendicular alignment observed when cell contractility is at normal levels for cells on silicone rubber coated with collagen (cf. Figs. 2A and 2C) or fibronectin [21]. In the case of a step stretch, cell and SF alignment was only observed on soft collagen gels, but not on silicone rubber coated with collagen (cf. Figs. 4E–F) ) or fibronectin (data not shown).

We previously reported a theoretical model predicting that SFs reorient perpendicular to the direction of cyclic stretch on matrix-coated silicone rubber to avoid excessive levels of tension acting on actomyosin binding sites [1], [4], [8], [9]. On relatively stiff silicone rubber sheets, where SF tension is already high under static conditions, the model predicts that tension is at an optimal level. Cyclic stretching at a high strain rate perturbs tension from this optimal level, promoting the disassembly of SFs and their preferred reassembly in an orientation perpendicular to the direction of stretch to minimize perturbations in tension. Since SF tension in cells on soft collagen gels is relatively low, we speculate that stretching increases tension toward the optimal level found in cells on a stiffer substrate, thereby promoting SF alignment parallel to the direction of stretch. The data herein provides the motivation for future development of our model to explicitly describe the role of substrate stiffness.

The dependence of alignment on cyclic stretch frequency is consistent with previous measurements using U2OS cells and endothelial cells on fibronectin-adsorbed silicone rubber [1], [5], [9]. In the present study, strain rate sensitivity was observed in cells cultured on collagen gels subjected to both steady and cyclic stretch patterns. Cyclic stretch at 0.01 and 1 Hz consisted of linear ramps (upward and downward) in strain of 0.2 and 20%/s, respectively. Cells subjected to steady stretch at a ramp rate of 20%/s showed significantly more cell and SF alignment, as compared to cells subjected to steady stretch at a ramp rate of 0.2%/s (cf. Figs. 4E–F). Our theoretical model predicts that the strain rate-dependence is due to the active regulation of SF tension by actomyosin sliding [4], [8]. Specifically, myosin II motors are predicted to translate along actin filaments in a direction that restores the forces acting on myosin heads to the values generated under static conditions. At high strain rates, we predict that myosin motors cannot respond quickly enough to regulate tension, while myosin can maintain tension nearly constant at low strain rates. We speculate that a similar mechanism regulates the strain-rate dependence observed for cells stretched on soft collagen gels.

Our results provide evidence that two mechanisms contribute to stretch-induced alignment on soft collagen gels. Prestretched collagen gels are expected to have anisotropic mechanical properties, with greater stiffness in the direction of stretch. The alignment of the cells and SFs along the direction of greater stiffness (cf. Fig. 4) is consistent with the alignment of cells on pillar arrays with anisotropic rigidity [25]. Applying the stretch after the cells have spread on the collagen gel induced a greater extent of alignment, however (cf. Fig. 4). Since cells subjected to steady stretch are expected to experience both the stretch stimulus as well as the anisotropic rigidity of the gel, these results suggest that the act of stretching provides an additional contribution to the alignment response beyond changing the mechanical properties to the gel. Further, the effectiveness of the stretch stimulus depends on both the rate (cf. Fig. 4) and duration (cf. Fig. 5) of strain.

It is interesting to speculate on why the stretch stimulus is only effective on soft collagen gels, but not on collagen-coated sheets (cf. Fig. 4). Previous theoretical and experimental studies have indicated that cells are only sensitive to matrix stiffness within a limited range near the stiffness of the cell [26], [27]. These models predict that cells cannot significantly deform substrates several orders of magnitude stiffer than the cells. Consistent with this prediction, cellular strains estimated from images of cells before and after a 10% step stretch indicate that cells deformed noticeably less on the soft collagen gels than on collagen-coated silicone rubber, suggesting that the cells are attenuating the stretching of the adjacent substrate (Supplemental Fig. S2 and Table S1). Perturbations in the stiffness of these substrates are therefore expected to be undetectable by the cells. Further, the silicone rubber substrate is elastic, hence does not stiffen upon stretching.

Cells in stretched 3-D collagen matrices are often elongated in parallel with the predominant alignment of collagen fibrils [28]. It has been suggested that the cells follow the collagen fibrils in a process termed contact guidance. In the present study, cells on collagen gels aligned along the direction of stretch without fibril alignment (cf. Fig. 2E), indicating that mechanical cues directly regulated cell and SF alignment.

Extracellular matrix geometry and topography at the nanoscale can impact cellular function [29], [30]. Atomic force microscopy imaging of collagen-coated silicone sheets indicate a relatively uniform surface [30]. Collagen in fibrillar networks, on the other hand, is non-uniform with relatively large spaces between fibrils for cell attachment, which is necessary for cell adhesion inside 3-D collagen gels. Gavara et al. [17] observed that cells cultured on the surface of fibrillar collagen gels spread and displayed similar patterns of traction force distribution as cells seeded on polyacrylamide substrates coated with monomeric collagen, suggesting that the fibrillar nature of the collagen gels did not obviously change cell adhesive behavior on the surfaces of collagen gels vs. collagen-coated substrates. It is expected that the area density and configuration of cell binding sites on monomeric collagens adhering to silicone rubber will differ from that on fibrillar collagen. Thus, it would be advantageous to repeat these studies in the future using collagen-coated silicone rubber sheets with different Young’s moduli to more directly assess the effects of stiffness.

A recent study by Pang et al. [31] involving subjecting smooth muscle cells to stretch in 3-D collagen matrices showed an early cell response to external mechanical signals before they were fully spread out. Specifically, they observed initial cellular alignment within 2 h of seeding cells and cells were completely aligned parallel to direction of stretch after 6 h. Alignment of collagen fibrils along the stretch direction was only observed at 6 h and was localized to the front of cell protrusions and attributed to the observed migration of cells parallel to the direction of stretch. In the system used Pang et al [31], the collagen hydrogel is only anchored at two ends, which generally leads to fibril alignment even in the absence of stretching due to the forces generated by contractile cells [32]. Our results suggest that cells respond to both stretch-induced changes in stiffness and the stretch itself as part of the initial response that occurs before any significant collagen remodeling has occurred. Further, the collagen hydrogels in our system were attached to stretch chamber on all sides other than the top free surface, which is expected to constrain any collagen remodeling that may occur at later times.

Our results indicate that stretch-induced SF alignment on soft collagen gels is dependent on MLCK, but not Rho-kinase. Rho-kinase and MLCK regulate central and peripheral SF populations, respectively [33]. SFs in cells stretched on collagen-coated silicone rubber contained central and peripheral populations of stress fibers, while mainly peripheral stress fibers were observed in cells on collagen gels (cf. Fig. 2). We have previously shown that cyclic uniaxial stretch induces the formation of actin fibers oriented parallel to the direction of stretch in cells treated with inhibitors of the Rho GTPase pathway and MLCK [5], [21]. In the present study with cells on collagen gels, cyclic stretch-induced actin fiber alignment parallel to the stretch direction was still observed upon Rho-kinase inhibition, but no alignment was observed upon MLCK inhibition (cf. Fig. 6). We observed that ML7 treatment led to complete attenuation of SFs, while some actin bundles were observed in cells treated with Y27632 and these were oriented in the direction of stretch. Further, these actin bundles were located at the cell periphery, consistent with previous reports that Y27632 only inhibits SFs located centrally, while ML7 inhibits SFs located at the cell periphery [5], [21], [34].

Our findings shed new light on experimental and theoretical observations by other groups on cells stretched on soft 2D and 3D substrates [35]–[37]. Consistent with our predictions, the theoretical models of McGarry and Deshpande [35], [38] predict that softer substrate do not provide sufficient tension for SF persistence, causing dissociation of SFs, while cells on a stiffer substrate are predicted to contain large amount of dominant SFs under optimal tension. Genin and Elson [36] showed that SFs in cells inside a 3D engineered tissue construct undergo retraction and subsequent reinforcement when subjected to stretch. Retraction response was observed for SFs in all directions, while reinforcement response was observed only in the stretch direction. The reinforcement response and alignment of SFs in stretch direction is consistent with our observation on 2D soft collagen gels. Krishnan et al. [37] and Trepat et al. [39] also reported cytoskeletal fluidization and reinforcement in cells subjected to stretch on soft polyacrylamide substrates [37], . However, we did not observe an obvious fluidization or retraction in SFs after a step increase in stretch in cells expressing GFP-actin (cf. Fig. 7).

Recent studies by Quinlan et al. [18] and Faust et al. [41] report that cells have attenuated alignment in response to stretch on soft polyacrylamide and soft silicone rubber substrates, respectively. Faust et al. [41] subjected cells to stretch at frequencies in the mHz range. In our current and past studies, we observed no alignment when stretching cells at a frequency of 10 mHz. Thus we predict that low strain rate due to low frequency cyclic stretching is not sufficient to induce alignment. Furthermore, polyacrylamide and silicone rubber are elastic, hence do not stiffen upon stretching. In the absence of the anisotropic changes in substrate rigidity, stretch alone may not be sufficient to stimulate alignment. Moreover, Quinlan et al. and Faust et al. used different cells type (porcine aortic valve interstitial cells and primary human umbilical cord fibroblasts, respectively) than we did, which may also contribute to the apparent discrepancies.

## Conclusion

In summary, our results clearly demonstrate that cells respond to applied strains in a manner dependent on substrate rigidity. Recent experiments employing high-resolution traction force microscopy on polyacrylamide substrates indicate that focal adhesions individually sample the substrate rigidity and that FAK/phosphopaxillin/vinculin signaling defines the rigidity range over which cells migrate toward regions of higher rigidity [42]. On the other hand, experiments performed on elastic pillar arrays interpreted with a phenomenological model based on active gel theory suggest that rigidity-sensing is mediated by a large-scale mechanism originating in the cytoskeleton rather than local sensing at the level of focal adhesions [43]. While our results are consistent with a large-scale mechanism involving the actin cytoskeleton and myosin motor proteins, we cannot rule out the role of focal adhesion proteins. Further studies are necessary to elucidate the molecular mechanism by which cells integrate applied strain and substrate rigidity to determine their morphological response.

## Supporting Information

Figure S1 **Silicone rubber chamber with collagen gel before (A) and after stretch (B).**
(TIF)

Figure S2 **Influence of substrate stiffness on cell elongation during stretch.** Representative images of U2OS cells before and after a 10% step stretch depicting cell elongation and change in cell length in the direction of stretching for cells cultured on collagen gels (A) and collagen coated silicone rubber sheets (B) (n = 3).
(TIF)

Table S1 **Influence of substrate stiffness on cell strains during stretch.**
(DOCX)

Video S1 **Live Cell Microscopy.** Time-lapse video depicting data in Figure 7.
(AVI)
