# Supplementary figures and images for: The Direction of Stretch-Induced Cell and Stress Fiber Orientation Depends on Collagen Matrix Stress
Source: PLoS One. 2014 Feb 24;9(2):e89592. doi: 10.1371/journal.pone.0089592 (PMC3933569; doi:10.1371/journal.pone.0089592)

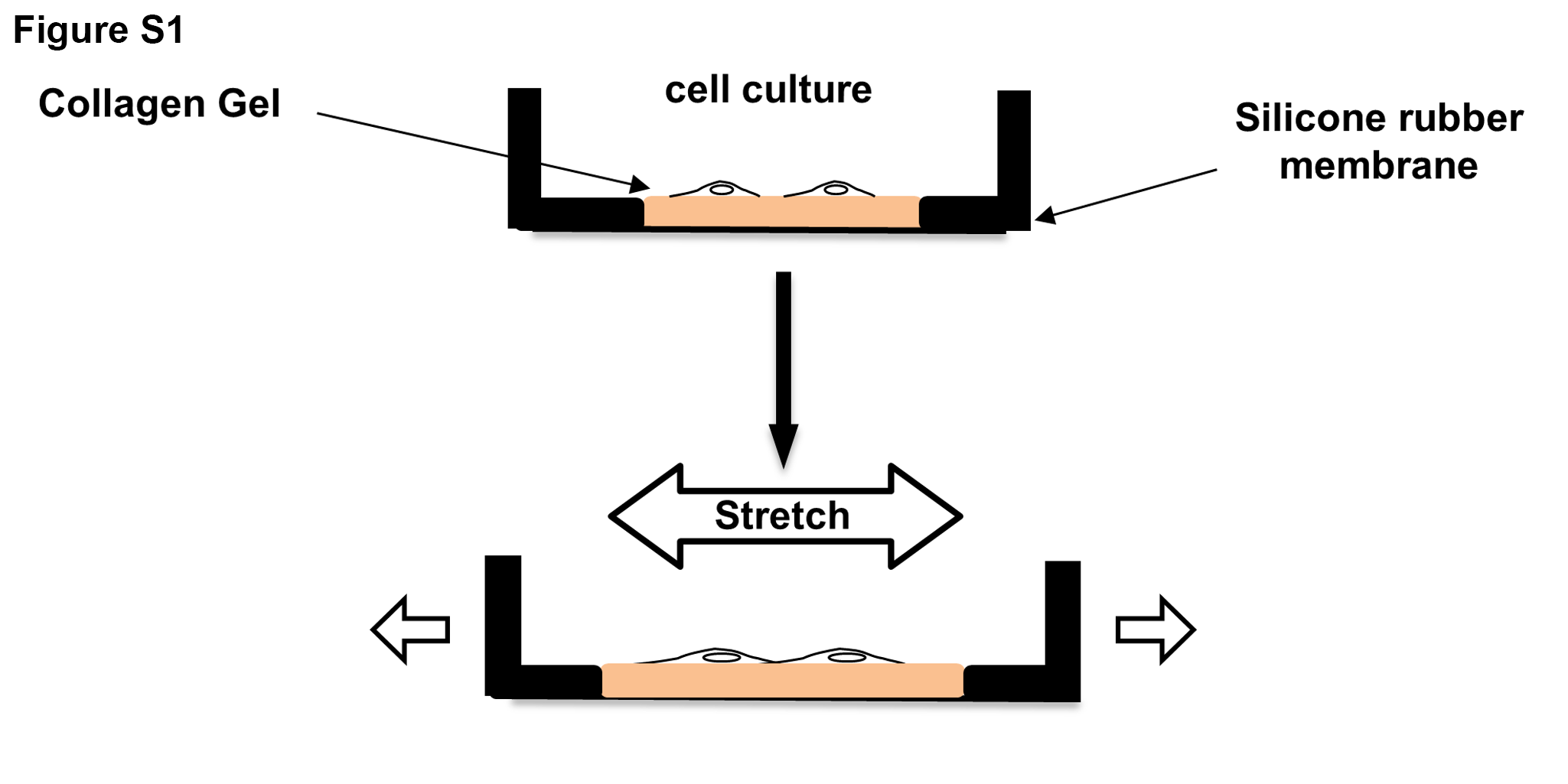

Supplement: Figure S1 — Silicone rubber chamber with collagen gel before (A) and after stretch (B). (TIF) [file pone.0089592.s001.tif]

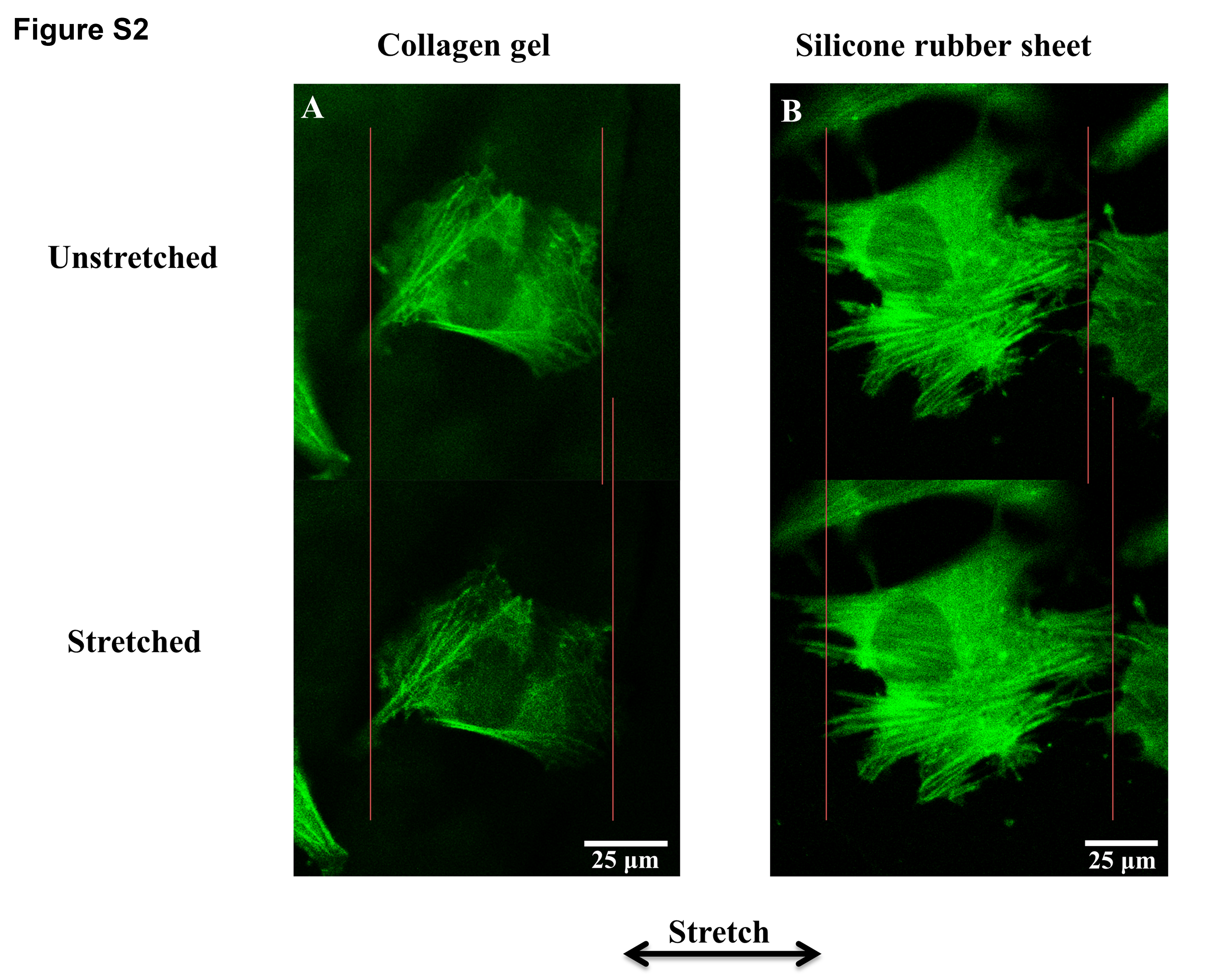

Supplement: Figure S2 — Influence of substrate stiffness on cell elongation during stretch. Representative images of U2OS cells before and after a 10% step stretch depicting cell elongation and change in cell length in the direction of stretching for cells cultured on collagen gels (A) and collagen coated silicone rubber sheets (B) (n = 3). (TIF) [file pone.0089592.s002.tif]
