# Supplementary material for: The Direction of Stretch-Induced Cell and Stress Fiber Orientation Depends on Collagen Matrix Stress
Source: PLoS One. 2014 Feb 24;9(2):e89592. doi: 10.1371/journal.pone.0089592 (PMC3933569; doi:10.1371/journal.pone.0089592)
Supplement: Table S1 — Influence of substrate stiffness on cell strains during stretch. (DOCX) [file pone.0089592.s003.docx]

**Table S1. Influence of substrate stiffness on cell strains during stretch**

|  |  | **Applied**  **Strain** | **Longitudinal Strain** | **Lateral**  **Strain** |
| --- | --- | --- | --- | --- |
| **Collagen** | Substrate alone | 0.1 | 0.093±0.009 | -0.040±0.01 |
|  | Cell 1 | 0.1 | 0.063±0.01 | -0.044±0.005 |
|  | Cell 2 | 0.1 | 0.058±0.014 | -0.054±0.017 |
|  | Cell 3 | 0.1 | 0.061±0.008 | -0.036±0.01 |
| **Silicone Rubber Sheet** | Substrate alone | 0.1 | 0.097±0.01 | -0.041±0.005 |
|  | Cell 1 | 0.1 | 0.091±0.013 | -0.046±0.004 |
|  | Cell 2 | 0.1 | 0.089±0.006 | -0.027±0.008 |
|  | Cell 3 | 0.1 | 0.098±0.089 | -0.024±0.004 |
